# Diagnostic utility of, and influence of tobacco usage and genetic predisposition on, immunoglobulin A, rheumatoid factor and anti-citrullinated peptide auto-antibodies in South African rheumatoid arthritis patients

**DOI:** 10.4314/ahs.v18i2.14

**Published:** 2018-06

**Authors:** Pieter Meyer, Mahmood Ally, Bridget Hodkinson, Ronald Anderson, Mohammed Tikly

**Affiliations:** 1 University of Pretoria, Immunology; NHLS, Immunology; 2 University of Pretoria, Internal Medicine; 3 University of the Witwatersrand, Rheumatology

**Keywords:** Tobacco usage, genetic predisposition, immunoglobulin A, anti-citrullinated peptide, South Africa, rheumatoid arthritis patients

## Abstract

**Background:**

The immunoglobulin A isotypes of anti-cyclic citrullinated peptide antibodies (ACPA) and rheumatoid factor (RF) are associated with disease severity and progression in Caucasian rheumatoid arthritis (RA) patients, as well as with genetic predisposition and tobacco use.

**Objectives:**

To compare levels of ACPA-IgA and RF-IgA with those of ACPA-IgG and cRF in a cohort of black South African RA patients and healthy controls.To investigate the relationship between IGA autoantibodies and disease severity, genetic predisposition and tobacco use

**Methods:**

RF-IgA and ACPA-IgA were determined in a cohort of predominantly black South African RA patients (n=75) in relation to serodiagnostic and prognostic potential, as well as tobacco use and genetic predisposition. Healthy control subjects were included to determine sensitivity, specificity and predictive values.

ACPA-IgG/IgA and RF-IgA were determined by enzyme immunoassay and hs-CRP and cRF by nephelometry. Cotinine levels were determined by ELISA.

**Results:**

The frequencies of ACPA-IgA and RF-IgA were 31% and 88% respectively compared to 88% for both types of traditional autoantibody procedures. ACPA-IgA was significantly higher (p=0.007) in patients with short disease duration, while linear regression analysis revealed a positive relationship with baseline disease activity scores. Levels of ACPA-IgG and ACPA-IgA were significantly higher in tobacco users who carried the HLA shared epitope.

**Conclusion:**

Although lacking in serodiagnostic superiority over cRF and ACPA-IgG, inclusion of RF-IgA and ACPA-IgA in autoantibody panels may provide insights into disease pathogenesis, interactions between tobacco usage and HLA genotype in the production of potentially disease-triggering ACPA-IgA antibodies.

## Introduction

Rheumatoid arthritis (RA) is a crippling disease characterized by chronic inflammation of the articular joints of patients leading to cartilage destruction and disease progression. The incidence is around 1–3% of the general population with a significantly higher prevalence in females. The inclusion of measurement of rheumatoid factor (RF) and anti-citrullinated peptide (ACPA) antibodies, generally composite RF (cRF) and ACPA-IgG, in the 2010 ACR/EULAR RA classification criteria is an indication of the sero-diagnostic utility of measurement of these auto-antibodies in RA^1^ and is well established worldwide^2^–^5^.

The profile and complex interplay of various RA-associated auto-antibodies and their different isotypes is influenced by the ethnic, genetic and environmental factors, with RF isotypes having been shown to be associated with a poor prognosis in Chinese and Indian, but not in Malay RF patients^6^, while in Caucasians, ACPA-IgA have been shown to be associated with severe disease and radiographic progression^11^,^12^. However, less is known about the diagnostic and prognostic potential of measurement of ACPA and RF auto-antibodies of the IgA isotype in other ethnicities with RA, as well as their associations with prominent disease risk factors, specifically tobacco use (i.e. cigarettes and snuff) and positivity for the human leucocyte antigen - shared epitope (HLA-SE).

These issues have been addressed in the current study in which the levels and prevalence ACPA-IgA and RF-IgA auto-antibodies have been compared with those of ACPA-IgG and cRF in a cohort of disease-modifying anti-rheumatic drug (DMARD)-naïve, black, predominantly female, South African patients with RA, and a control group of healthy individuals, the findings of which may be broadly applicable to other populations.Additional aims included investigation of the relationships of these IgA auto-antibodies with: i) disease severity as measured by the simplified disease activity score (SDAI); and ii) genetic predisposition and tobacco use. As mentioned above these potentially important issues have not been investigated previously in our geographic region.

## Patients & methods

The study was approved by the Research Ethics Committees of the Faculties of Health Sciences of the Universities of the Witwatersrand and Pretoria and conformed to good clinical and laboratory practice and the Helsinki declaration. Patients were recruited from the Chris Hani Baragwanath Academic hospital (CHBAH, Soweto, South Africa) and Steve Biko Academic Hospital (SBAH, Pretoria, South Africa) Rheumatology Out-patient clinics. Ethics certificate reference numbers: 09/2017 (UP) and M150513 (WITS).

Informed consent, signed by each patient, was explained and monitored by the attending physician. Although the informed consent document was available only in English, a translator was present at both the CHBAH and SBAH Rheumatology clinics to ensure that patients fully understood the nature of their participation in the study. No financial compensation was offered, and it was explained to the participants that the results of this study may lead to better understanding of their disease. Healthy controls where anonymously sourced from the South African Blood Transfusion Service.

This study was a retrospective, cross-sectional study the primary objective of which was to determine the utility of measurement of immunoglobin A (IgA) rheumatoid factor (RF) and anti-cyclic citrullinated peptide antibodies (ACPA) in sero-diagnosis and prognosis of rheumatoid arthritis (RA), as well as their relationships with known risk factors and roles as predictive biomarkers of disease severity in black South African patients with RA. To our knowledge, the study is the first of its type undertaken in sub-Saharan Africa. The study cohort consisted of a sub-group (n=75) of black African patients that formed part of a previously described cohort^7^, all diagnosed with RA according to the 1987 ACR RA criteria, all of whom were DMARD-naïve at baseline. Methods for serological measurement cRF and ACPA-IgG have been described previously for this cohort of RA patients^7^–^9^.

Twenty healthy individuals were used as controls, matched in so far as possible for age, ethnicity and gender to determine if commercial stated cut-off values for RF and ACPA IgA are valid, to assist in calculating specificity, sensitivity and positive/ negative predictors and cotinine levels to determine tobacco use cut-offs.

Venous blood was collected in 2x5ml test tubes, 1 with added clotting factor and 1 containing the anti-coagulant, EDTA. The serum tubes were left to clot and centrifuged thereafter for 10 minutes at 2000 × g. The serum was removed, aliquoted and stored at −20°C until analyzed. The EDTA anti-coagulated blood was used the extract whole genomic DNA using the Maxwell Promega automated DNA extraction instrument and Promega Maxwell® 16 Tissue DNA purification kits (Promega Corporation, Madison, USA).

Clinical disease activity was recorded using the standard classification for SDAI; remission = ≤ 3, low disease activity (LDA) = >3 − ≤11, mild disease activity (MDA) = >11 - ≤26 and high disease activity (HDA) >26^12^.

EliA ACPA-IgA and RF-IgA were measured using fluorescence enzyme immunoassay (FEIA) on the automated Phadia 250 platform (Phadia AB, Uppsala, Sweden). Cut-off values to determine positivity for the individual auto-antibodies were as follows: >10 units/millilitre (U/ml) serum for EliA ACPA-IgG and ACPA-IgA, >20 IU/ml for EliA RF IgA, >15 U/ml for cRF. High-sensitivity serum C-reactive protein (CRP) concentrations were measured by latex-enhanced laser nephelometry (Siemens South Africa, Midrand, South Africa) and the results expressed as micrograms (µg)/ml.

Cotinine was determined using an ELISA kit (Calbiotech Inc., Spring Valley, CA, USA). The results are expressed as nanograms (ng/ml). A cut-off above 10 ng/ml was taken as indicative of active tobacco use as recommended by the product insert.

The molecular procedures used for HLA-DRB1 typing and HLA-SE categorization have been described previously^8^. Briefly, purity and concentrations of the extracted DNA were determined, and molecular analysis carried out using a high-resolution rSSO typing technique utilising Luminex® bead technology for HLA-DRB1 alleles (LABType HD Class II DRB1 Typing Test, One-Lambda, Thermo Fisher Scientific, USA) and alleles assigned using One Lambda Fusion software. The HLA-SE has been classified and validated according to the RAA amino acid sequence at positions 72–74 of the third hypervariable region of the HLA-DRB1 gene and assigned SS if both HLA-DRB1 alleles express the RAA motif, or SX if only one HLA-DRB1 allele expresses the RAA motif. Further classification of the SE-sequence beyond the RAA amino acid sequences at positions 72 to 74 have a modulatory effect (positions 70 and 71). Glutamine (Q) or arginine (R) at position 70 of the 3^rd^ hypervariable region of the HLA-DRB1 gene conferred a high risk of severe disease, while aspartic acid (D) is associated with low risk. Lysine (K) at position 71 was indicative of the highest risk, while arginine (R) alanine (A) or glutamic acid (E) were classified as low risk^10^.

### Expression of results and statistical analysis

Results are expressed as the median values with interquartile ranges. Descriptive and inferential statistics techniques were used in the analyses. Tests for association of contingency tables were performed using two-tailed Chi2 tests. One-way ANOVA was performed using the Dunn and Kruskal-Wallis tests for non-parametric data for more than 2 groups, or the Mann-Whitney test when 2 groups were compared with Bonferroni correction where applicable. Correlation coefficients were derived from non-parametric Spearman's rank correlation test and correlation matrices using the Sidak's method p value correction for multiple testing. Statistical significance was determined by p-value <0.05 and confidence intervals of 95%. The analyses were done using STATA^13^.

The raw data is stored electronically for 15 years as per ethical requirement and is accessible by request to the corresponding author.

## Results

### Demographics

The demographics of the group of RA patients, as well as seropositivity rates for the biomarkers and SE-related data are summarized in Table 1.

**Table 1:** Patient demographics, disease indices, tobacco use, HLA-SE categories and autoantibody positivity rates in the group of RA patients

n=75	N (%)	Median iqr	(min-max)
Age(years)		47.8 19	(18 –75)
Females	61 (81)		
Males	14 (19)		

Disease Duration (months)		8.5 11.9	(2 –25)
<6 months	24 (32)		
6 to 12 months	27 (36)		
>12 months	24 (32)		

SDAI		40 25	(5 –76)
LDA, n(%)	2 (3)		
MDA, n(%)	17 (23)		
HDA, n(%)	56 (75)		

X-ray Score		1913	(1–48)
Erosions, n(%)	31 (47)		

Tobacco use	28 (37)		
HLA-SE	71 (95)		
Homozygous	41 (55)		
Heterozygous	30 (40)		

hs-CRP(mg/dl)		13 22	(1 –113)

RF(UI/ml)		161 403	(4 –1720)
RF *positivity*	65 (87)		
RF-IgA (IU/ml)		4154	(3–436)
RF *positivity*	66 (88)		
ACPA(U/ml)		494839	(2 –1621)
ACPA *positivity*	65 (88)		
ACPA-IgA(U/ml)		1122	(1–87)
ACPA-*IgA positivity*	23 (31)		

The sensitivities, specificities, positive predictive values (PPVs), negative predictive values (NPVs), prevalence rates and Likelihood Ratios for cRF, ACPA-IgG, RF-IgA and ACPA-IgA in the group of RA patients are shown in Table 2. These demonstrate that cRF and ACPA are the best overall predictors of disease. ACPA-IgA antibodies, on the other hand, have the best specificity and PPV of the group of biomarkers, but show lesser sensitivity and NPV in comparison with cRF and ACPA. Likelihood ratios for cRF and ACPA rank the highest with cRF at 9.32. Importantly, RF-IgA and ACPA-IgA were inferior to the traditional cRF and ACPA-IgG sero-diagnostic biomarkers recommended by the ACR 2010.

**Table 2:** Sensitivities, Specificities, PPVs, NPVs and Likelihood Ratios for the various auto antibodies

	Sensitivity % (95CI)	Specificity % (95CI)	PPV % (95CI)	NPV % (95CI)	Likelihood Ratio
cRF	86.7 (76.8 –93.4)	90.7 (82.5– 95.9)	89.0 (79.5– 95.2)	88.6 (80.1– 94.4)	9.32
ACPA	86.7 (76.8 –93.4)	86.0(77.3– 92.3)	83.3 (73.2 – 90.8)	88.9 (80.5– 94.5)	6.19
RF-IgA	50.0 (33.8–66.2)	58.2(32.9– 81.6)	74.1 (53.7– 88.9)	33.3 (17.2 – 52.8)	1.20
ACPA-IgA	30.7 (20.5 – 42.4)	95.0 (75.1 – 99.9)	95.8 (78.9 – 99.9)	26.8 (16.9 – 38.6)	1.76

Highest results achieved are in BOLD

**Figure 1:**
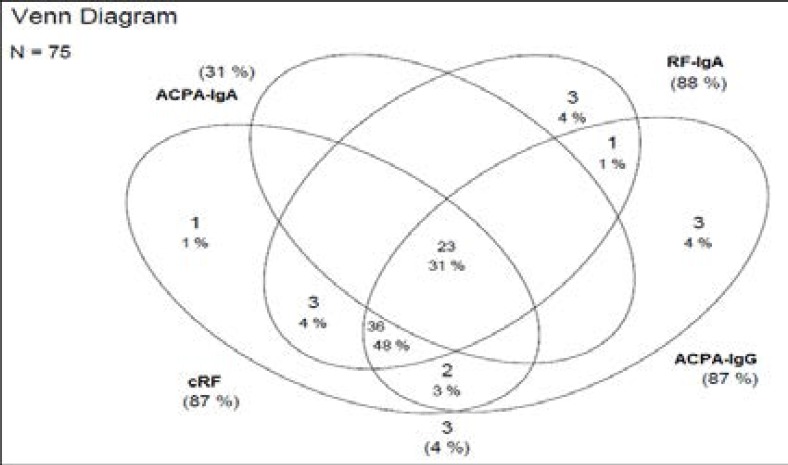
Venn diagram of positive reactivity patterns of ACPA-IgG, cRF, ACPA-IgA RF-IgA individually as well as cross-reactivity patterns between these biomarkers. The Venn diagram indicates that 31% (n=23) of RA patients had cRF and ACPA autoantibodies of both serotypes (IgG and IgA), while 48% (n=36) were positive for ACPA-IgG, cRF and RF-IgA, but not for ACPA-IgA.

The overall combination of reactivities and cross-reactivities of all the auto-antibodies are demonstrated in a Venn diagram as Figure 1.

The healthy control subjects had median values (iqr) of 16 (53) IU/ml and 2 (3) IU/ml for RF-IgA and ACPA-IgA, respectively. RF-IgA seropositivity amongst the healthy control subjects was 41% (95% CI: 19% – 67%), while ACPA-IgA demonstrated a positivity rate of 18% (95% CI: 5%–46%).

### Disease severity (SDAI scores & disease duration at baseline)

Duration since onset of symptoms in months, SDAI scores and the presence of erosions at presentation were used as markers of disease severity. At baseline, 25% (n=19) of patients were scored as MDA and 75% (n=56) as HDA, while no patients were in remission or LDA. Linear regression analysis revealed a statistically significant positive relationship between ACPA-IgA and SDAI at baseline (p=0.030, R2=0.0646, β=0.3, α=6.1, CI: 0.03 – 0.58).

### Risk factors

#### Tobacco use

Tobacco users (serum cotinine >10ng/ml) presented with a higher SDAI score at baseline than non-users (p=0.036) using the Mann-Whitney U-test. ACPA -IgA levels were higher in tobacco users than non-users (p=0.007) in the group of patients with disease duration of <12 months, but not in those with disease duration >12 months.

#### HLA-SE

Table 1 indicates that 71 (95%) patients had an HLASE allele, of which 30 (40%) were heterozygous (SX) i.e. had 1 of the SE-associated alleles and 55% where homozygous (SS) for HLA-SE. Only 4 (5%) of the patients expressed no HLA-SE phenotype (XX) in keeping with previously described findings in this cohort of patients^8^. This showed that the 71 patients with HLA-SE alleles could be categorized as High Risk (HR) and Low Risk (LR), according to the amino acids at position 70 and 71. The HR and LR groups consisted of 48 (68%) and 23 (31%) patients respectively. A further subdivision consisting of homozygous, heterozygous, LR and HR HLASE allele permutations revealed the following groupings: i) 23 (31%) patients had both the LR and HR HLA-SE alleles (DUAL), ii) 10 (13%) were homozygous for the HR alleles (HoHR), iii) 17 (23%) were heterozygous for the HR alleles (HeHR), iii) 8 (11%) were homozygous for the LR alleles (HoLR), iv) 13 (17%) were heterozygous for the LR alleles (HeLR), and v) 4(5%) had no HLA-SE alleles.

**Figure 2:**
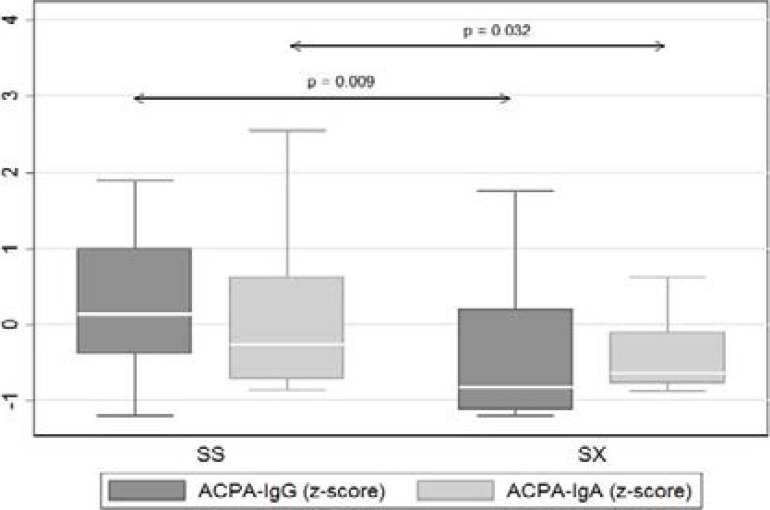
Median values of ACPA, and ACPA-IgA in patients categorised according to HLA-SE homozygosity (SS) and heterozygosity (SX). Values were standardised (z-scores) to display on common y-axis (standardisation: x* = (x-m)/SD Where m is the mean of x, and SD is the standard deviation of x).

As shown in Figure 2, statistically significant differences in serum levels of ACPA-IgG (p = 0.009) and ACPA-IgA (p=0.032) were noted when comparing the SS vs. SX HLA-SE allele expression groups, with the homozygous (SS) patients having consistently higher median autoantibody titres than their heterozygous (SX) counterparts. This was not apparent in the cases of both cRF and RF-IgA.

As shown in Figure 3A, the median titers of ACPA-IgA auto-antibodies in tobacco users show a trend, albeit insignificant (p=0.078), towards higher values, while the data shown in Figure 3B indicate an association between HLA-SE homo- and heterozygosity, tobacco use and ACPA-IgA, which reaches statistical significance only in the heterozygous group ( p=0.028). This may be explained by the presence of the HR modulatory gene present in 57% (n=17) present in the SX group, whereas in the SS group only 24% (n=10) of patients have the HR associated amino acid sequence. The majority 56% (n=23) of the SS patients express the LR modulatory gene in combination with the HR gene, with a further 20% (n=8) homozygous for LR. These findings are in agreement with the involvement of the HR HLA-SE genes in the immunopathogenesis of RA, potentiated by tobacco use.

**Figure 3A:**
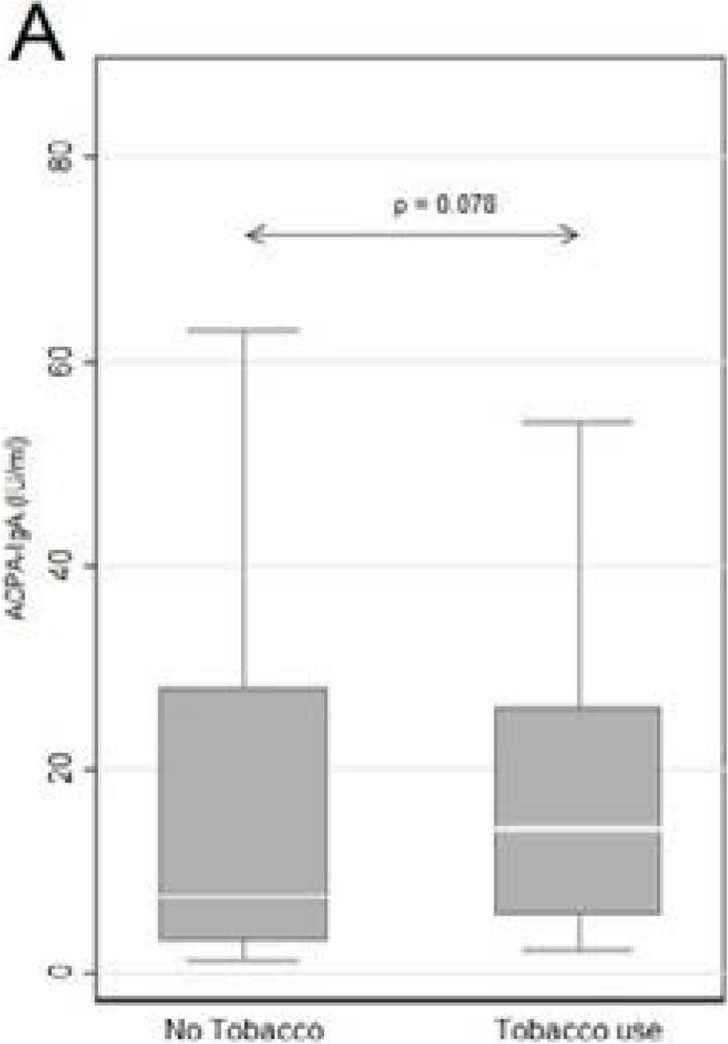
Median ACPA-IgA values in RA tobacco users and non-users showing a trend towards higher ACPA-IgA titres in users of tobacco.

**Figure 3B:**
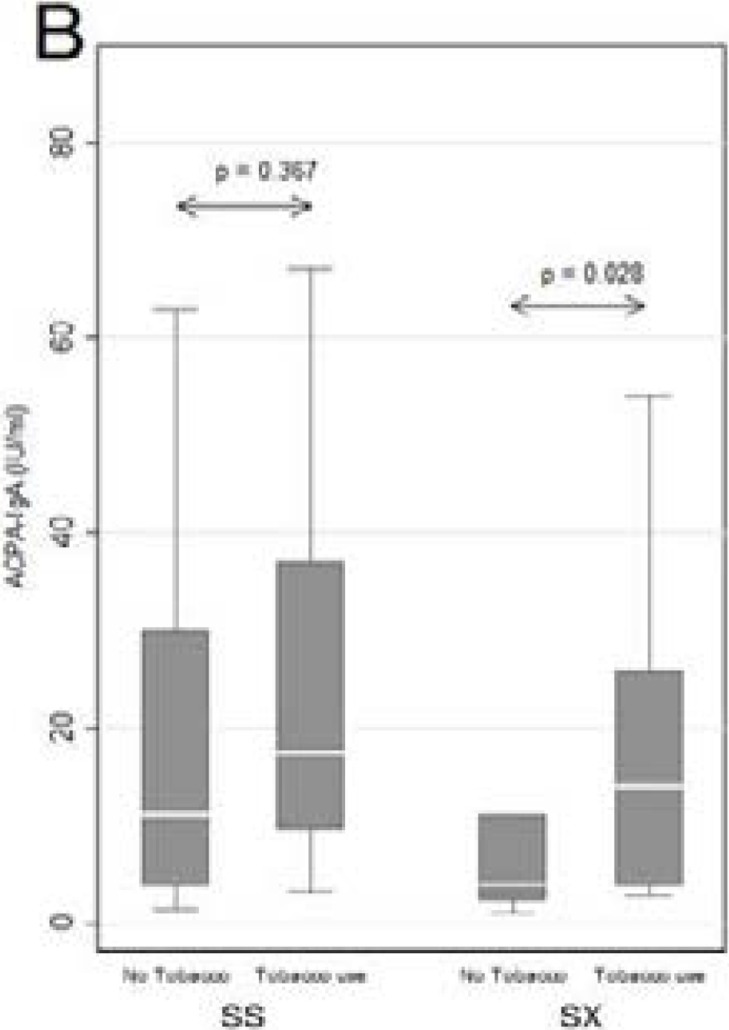
Median ACPA-IgA values in tobacco users and non-users categorised according to Shared Epitope homozygosity (SS) and heterozygosity (SX).

## Discussion

The current study demonstrates a lack of diagnostic or predictive value of measurement of RF-IgA relative to that of cRF in this study group of DMARD-naïve, African RA patients. Measurement of ACPA-IgA, on the other hand, demonstrated an extremely high specificity and positive predictive value, as well as a positive association with disease activity, but was notably lacking in both sensitivity and negative predictive value in comparison with both ACPA-IgG and cRF. Assuming that these observations can be extrapolated to other population groups, they appear to argue against the clinical utility of measurement of either RF-IgA or ACPA-IgA as diagnostic adjuncts to cRF and ACPA^11^,^12^.

Interestingly, however, tobacco usage, which was associated with higher disease activity on presentation, was also associated with higher levels of ACPA-IgA, specifically in those RA patients categorized as having disease duration of <12 months. Higher levels of ACPA-IgA prior to disease onset may be a reflection of epitope spreading and isotype expansion, a phenomenon well described in RA^13^ In addition, when comparing RA tobacco users with RA non-tobacco users, a statistically significant, positive association between smoking, SE genotype and serum concentrations of ACPA-IgA was also evident^14^–^16^. These observations are of possible significance in relation to the role of smoking and HLA genotype in the immunopathogenesis of RA, seemingly strengthening the link with smoking-related protein citrullination in the airway mucosa^17^–^19^, and possibly the gastrointestinal mucosa^20^,^21^, resulting in interactions of citrullinated proteins with SE-expressing T lymphocytes, leading to the production of potentially pathogenic ACPA-IgA auto-antibodies^13^. In this context, it is, however, noteworthy, that tobacco usage in the current cohort of RA patients is predominantly inhalation of powdered tobacco (snuff), as opposed to active smoking, with both types of tobacco usage resulting in equivalent circulating concentrations of cotinine^22^, suggesting, that like active smoking, usage of smokeless tobacco products, particularly those that are inhaled, may also predispose to development of RA.

Notwithstanding the relatively small number of patients, which is a limitation of this study, it is, however, noteworthy that similar sized or smaller cohort studies have been published in a number of peer-reviewed journals as illustrated in the systematic review article by Peter Taylor et al.^22^ The findings of our study do, however, appear to indicate the lack of superiority of RF-IgA and ACPA-IgA over cRF and ACPA-IgG in the sero-diagnosis of RA. On the other hand, the finding of associations of ACPA-IgA with tobacco usage and HLA genotype indicates that this augmentative combination of risk factors for development and severity of RA in Black South Africans is similar to that described for their counterparts of Caucasian origin. Clearly, usage of any type of tobacco product is to be discouraged in those identified as being at high risk for development of RA, or who are in the early stages of the disease.
